# Tetra­aqua­bis­[4-(1*H*-imidazol-1-yl-κ*N* ^3^)benzoato]cobalt(II)

**DOI:** 10.1107/S1600536812010562

**Published:** 2012-03-28

**Authors:** Jian Guo, Shao-Wei Tong, Jian-She Liu, Wen-Dong Song, Jing-Bo An

**Affiliations:** a College of Chemistry and Chemical Engineering, Donghua University, Shanghai 200051, People’s Republic of China; b College of Food Science and Technology, Guangdong Ocean University, Zhanjiang 524088, People’s Republic of China; c College of Science, Guangdong Ocean University, Zhanjiang 524088, People’s Republic of China

## Abstract

In the title compound, [Co(C_10_H_7_N_2_O_2_)_2_(H_2_O)_4_], the Co^II^ atom lies on an inversion centre and displays a slightly distorted octa­hedral geometry. The coordination sphere is defined by two mutually *trans* N atoms from two 4-(imidazol-1-yl)benzoate ligands and the O atoms from four water mol­ecules. The crystal structure is stabilized by O—H⋯O hydrogen bonds.

## Related literature

For our previous work on imidazole derivatives as ligands, see: Li, Song *et al.* (2011 ▶); Li, Ma *et al.* (2011 ▶); Fan *et al.* (2010 ▶); Li *et al.* (2010 ▶).
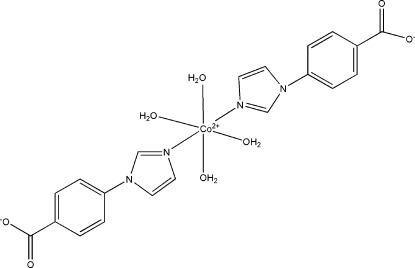


## Experimental

### 

#### Crystal data

[Co(C_10_H_7_N_2_O_2_)_2_(H_2_O)_4_]
*M* *_r_* = 505.35
Monoclinic, 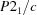

*a* = 12.1976 (15) Å
*b* = 10.6555 (13) Å
*c* = 7.9602 (10) Å
β = 96.816 (2)°
*V* = 1027.3 (2) Å^3^
*Z* = 2
Mo *K*α radiation
μ = 0.89 mm^−1^
*T* = 296 K
0.22 × 0.19 × 0.15 mm

#### Data collection

Bruker APEXII area-detector diffractometer
Absorption correction: multi-scan (*SADABS*; Sheldrick, 1996 ▶) *T*_min_ = 0.616, *T*_max_ = 0.744
7094 measured reflections
1850 independent reflections
1720 reflections with *I* > 2σ(*I*)
*R*_int_ = 0.048

#### Refinement

*R*[*F* ^2^ > 2σ(*F* ^2^)] = 0.032
*wR*(*F* ^2^) = 0.092
*S* = 1.07
1850 reflections
151 parameters
6 restraints
H-atom parameters constrained
Δρ_max_ = 0.31 e Å^−3^
Δρ_min_ = −0.36 e Å^−3^

### 

Data collection: *APEX2* (Bruker, 2004 ▶); cell refinement: *SAINT* (Bruker, 2004 ▶); data reduction: *SAINT*; program(s) used to solve structure: *SHELXS97* (Sheldrick, 2008 ▶); program(s) used to refine structure: *SHELXL97* (Sheldrick, 2008 ▶); molecular graphics: *XP* in *SHELXTL* (Sheldrick, 2008 ▶); software used to prepare material for publication: *SHELXL97*.

## Supplementary Material

Crystal structure: contains datablock(s) I, global. DOI: 10.1107/S1600536812010562/sj5202sup1.cif

Structure factors: contains datablock(s) I. DOI: 10.1107/S1600536812010562/sj5202Isup2.hkl

Additional supplementary materials:  crystallographic information; 3D view; checkCIF report

## Figures and Tables

**Table 1:** Selected bond lengths (Å)

Co1—O1*W*	2.1286 (13)
Co1—O2*W*	2.0644 (13)
Co1—N2	2.1238 (15)

**Table 2:** Hydrogen-bond geometry (Å, °)

*D*—H⋯*A*	*D*—H	H⋯*A*	*D*⋯*A*	*D*—H⋯*A*
O2*W*—H3*W*⋯O2^i^	0.84	1.88	2.6745 (17)	157
O2*W*—H4*W*⋯O2^ii^	0.85	1.86	2.6964 (18)	170
O1*W*—H2*W*⋯O2^i^	0.83	2.03	2.8287 (19)	163
O1*W*—H1*W*⋯O1^iii^	0.83	1.87	2.7014 (18)	177

Symmetry codes: (i) 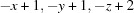; (ii) 
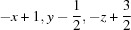
; (iii) 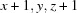.

**Fig. 1.**
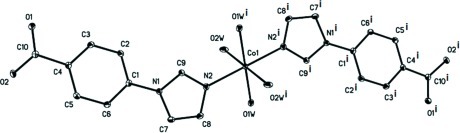
The structure of the title compound, showing the atomic numbering scheme. Non-H atoms are shown with 30% probability displacement ellipsoids (H atoms are omitted for clarity). [Symmetry code: i = 2 - x, 1 - y, 2 - z.]

## supplementary crystallographic information

### Comment

During the past decade, considerable efforts have been devoted to design and construct new metal-organic frameworks due to their intriguing structural diversity and potential application in many areas. In recent years, our research group has shown great interest in the design and synthesis of interesting metal-organic complexes with imidazole derivatives such as 2-propyl-imidazole-4,5-dicarboxylic acid (Fan *et al.*, 2010; Li *et al.*, 2010) and 2-ethyl-1*H*-imidazole-4,5-dicarboxylic acid (Li, Song *et al.*, 2011; Li, Ma *et al.*, 2011). In this paper, we report the synthesis and structure of a new Co^II^ complex, [Co(C_10_H_7_N_2_O_2_)_2_(H_2_O)_4_].

As illustrated in Fig. 1, the title compound, consists of a Co^II^ cation, two deprotonated 4-(imidazol-1-yl)benzoic acid ligands and four coordinated water molecules. Each six coordinate Co^II^ ion, lies on an inversion center with the N atoms of the 4-(imidazol-1-yl)benzoic acid ligands mutually *trans* and the four water molecules in an equatorial plane. The Co–N distance is 2.1238 (15) Å and Co–O distances are 2.0643 (13) Å and 2.1287 (14) Å, respectively. It is interesting to note that in this molecule, the 4-(imidazol-1-yl)benzoic acid ligands coordinate to Co(II) *via* a nitrogen atom of the imidazole residue unlike several other complexes of dicarboxylic acid derivatives we have reported previously, which coordinate to metal atoms via the carboxylate group (Fan *et al.*, 2010; Li *et al.*, 2010; Li, Song *et al.*, 2011; Li, Ma *et al.*, 2011). In the crystal structure, molecules form a three-dimensional network through an extensive series of intermolecular O—H···O hydrogen bonds.

### Experimental

A mixture of Co(NO_3_)_2_.6H_2_O (0.5 mmol, 0.15 g) and 4-(imidazol-1-yl)benzoic acid (1 mmol, 0.19 g) in 12 ml of H_2_O was sealed in an autoclave equipped with a Teflon liner (20 ml) and then heated to 433 K for 4 days. After gradual cooling to room temperature, red crystals were obtained and collected by filtration with a yield of 31% based on Co.

### Refinement

Carbon and nitrogen bound H atoms were placed at calculated positions and were treated as riding on the parent C or N atoms with C—H = 0.93 Å, N—H = 0.86 Å, and with *U*_iso_(H) = 1.2 *U*_eq_(C, N). H atoms of the water molecules were located in a difference Fourier map and refined as riding with an O—H distance restraint of 0.84 (1) Å and with *U*_iso_(H) = 1.5 *U*_eq_. The H···H distances within the water molecules were also restrained to 1.39 (1) Å.

### Crystal data

**Table:**

[Co(C_10_H_7_N_2_O_2_)_2_(H_2_O)_4_]	*F*(000) = 522
*M**_r_* = 505.35	*D*_x_ = 1.634 Mg m^−^^3^
Monoclinic, *P*2_1_/*c*	Mo *K*α radiation, λ = 0.71073 Å
Hall symbol: -P 2ybc	Cell parameters from 5837 reflections
*a* = 12.1976 (15) Å	θ = 2.8–27.9°
*b* = 10.6555 (13) Å	µ = 0.89 mm^−^^1^
*c* = 7.9602 (10) Å	*T* = 296 K
β = 96.816 (2)°	Block, red
*V* = 1027.3 (2) Å^3^	0.22 × 0.19 × 0.15 mm
*Z* = 2	

### Data collection

**Table:**

Bruker APEXII area-detector diffractometer	1850 independent reflections
Radiation source: fine-focus sealed tube	1720 reflections with *I* > 2σ(*I*)
Graphite monochromator	*R*_int_ = 0.048
φ and ω scan	θ_max_ = 25.2°, θ_min_ = 1.7°
Absorption correction: multi-scan (*SADABS*; Sheldrick, 1996)	*h* = −14→14
*T*_min_ = 0.616, *T*_max_ = 0.744	*k* = −12→12
7094 measured reflections	*l* = −9→9

### Refinement

**Table:**

Refinement on *F*^2^	Primary atom site location: structure-invariant direct methods
Least-squares matrix: full	Secondary atom site location: difference Fourier map
*R*[*F*^2^ > 2σ(*F*^2^)] = 0.032	Hydrogen site location: inferred from neighbouring sites
*wR*(*F*^2^) = 0.092	H-atom parameters constrained
*S* = 1.07	*w* = 1/[σ^2^(*F*_o_^2^) + (0.0491*P*)^2^ + 0.4159*P*] where *P* = (*F*_o_^2^ + 2*F*_c_^2^)/3
1850 reflections	(Δ/σ)_max_ < 0.001
151 parameters	Δρ_max_ = 0.31 e Å^−^^3^
6 restraints	Δρ_min_ = −0.36 e Å^−^^3^

### Special details

**Table:**

Geometry. All e.s.d.'s (except the e.s.d. in the dihedral angle between two l.s. planes) are estimated using the full covariance matrix. The cell e.s.d.'s are taken into account individually in the estimation of e.s.d.'s in distances, angles and torsion angles; correlations between e.s.d.'s in cell parameters are only used when they are defined by crystal symmetry. An approximate (isotropic) treatment of cell e.s.d.'s is used for estimating e.s.d.'s involving l.s. planes.
Refinement. Refinement of *F*^2^ against ALL reflections. The weighted *R*-factor *wR* and goodness of fit *S* are based on *F*^2^, conventional *R*-factors *R* are based on *F*, with *F* set to zero for negative *F*^2^. The threshold expression of *F*^2^ > σ(*F*^2^) is used only for calculating *R*-factors(gt) *etc*. and is not relevant to the choice of reflections for refinement. *R*-factors based on *F*^2^ are statistically about twice as large as those based on *F*, and *R*- factors based on ALL data will be even larger.

### Fractional atomic coordinates and isotropic or equivalent isotropic displacement parameters (Å^2^)

**Table:**

	*x*	*y*	*z*	*U*_iso_*/*U*_eq_	
C1	0.55457 (14)	0.61139 (17)	0.8770 (2)	0.0122 (4)	
C2	0.52906 (16)	0.51963 (18)	0.7536 (2)	0.0159 (4)	
H2	0.5827	0.4630	0.7282	0.019*	
C3	0.42300 (17)	0.51351 (17)	0.6690 (3)	0.0158 (4)	
H3	0.4066	0.4530	0.5855	0.019*	
C4	0.34080 (14)	0.59535 (17)	0.7058 (2)	0.0122 (4)	
C5	0.36592 (15)	0.68373 (17)	0.8335 (2)	0.0146 (4)	
H5	0.3111	0.7374	0.8625	0.018*	
C6	0.47249 (14)	0.69250 (17)	0.9181 (2)	0.0140 (4)	
H6	0.4887	0.7526	1.0022	0.017*	
C7	0.70472 (16)	0.70111 (19)	1.0893 (2)	0.0208 (4)	
H7	0.6644	0.7599	1.1427	0.025*	
C8	0.81424 (16)	0.67658 (18)	1.1233 (2)	0.0194 (4)	
H8	0.8623	0.7168	1.2056	0.023*	
C9	0.75273 (15)	0.55233 (18)	0.9212 (2)	0.0156 (4)	
H9	0.7490	0.4911	0.8374	0.019*	
C10	0.22864 (14)	0.59036 (17)	0.6021 (2)	0.0132 (4)	
Co1	1.0000	0.5000	1.0000	0.01069 (15)	
N1	0.66473 (12)	0.62083 (14)	0.95870 (17)	0.0124 (3)	
N2	0.84354 (12)	0.58317 (15)	1.01756 (18)	0.0150 (3)	
O1	0.22401 (10)	0.54154 (14)	0.45864 (16)	0.0187 (3)	
O2	0.14737 (10)	0.63844 (12)	0.66430 (15)	0.0158 (3)	
O1W	1.02638 (11)	0.52252 (13)	1.26744 (17)	0.0167 (3)	
H1W	1.0866	0.5265	1.3281	0.025*	
H2W	0.9794	0.4800	1.3089	0.025*	
O2W	0.93348 (12)	0.32615 (13)	1.04136 (16)	0.0231 (3)	
H4W	0.9150	0.2686	0.9701	0.035*	
H3W	0.8941	0.3241	1.1211	0.035*	

### Atomic displacement parameters (Å^2^)

**Table:**

	*U*^11^	*U*^22^	*U*^33^	*U*^12^	*U*^13^	*U*^23^
C1	0.0122 (8)	0.0135 (9)	0.0108 (8)	−0.0007 (7)	0.0013 (6)	0.0028 (7)
C2	0.0131 (10)	0.0182 (9)	0.0161 (10)	0.0045 (7)	0.0010 (8)	−0.0030 (7)
C3	0.0163 (10)	0.0177 (10)	0.0130 (9)	0.0001 (7)	−0.0007 (8)	−0.0029 (7)
C4	0.0123 (9)	0.0147 (9)	0.0100 (8)	−0.0002 (7)	0.0023 (7)	0.0032 (7)
C5	0.0142 (9)	0.0144 (9)	0.0157 (9)	0.0027 (7)	0.0035 (7)	0.0003 (7)
C6	0.0153 (9)	0.0137 (9)	0.0132 (9)	−0.0011 (7)	0.0025 (7)	−0.0014 (6)
C7	0.0175 (10)	0.0228 (10)	0.0208 (10)	0.0045 (8)	−0.0025 (8)	−0.0110 (8)
C8	0.0170 (10)	0.0222 (10)	0.0179 (9)	0.0011 (8)	−0.0034 (7)	−0.0074 (8)
C9	0.0118 (9)	0.0203 (10)	0.0145 (9)	0.0017 (7)	0.0011 (7)	−0.0035 (7)
C10	0.0145 (9)	0.0132 (9)	0.0119 (9)	−0.0004 (7)	0.0020 (7)	0.0039 (7)
Co1	0.0086 (2)	0.0135 (2)	0.0097 (2)	−0.00079 (11)	0.00011 (15)	−0.00126 (11)
N1	0.0111 (7)	0.0148 (7)	0.0111 (7)	0.0016 (6)	0.0010 (6)	−0.0004 (6)
N2	0.0123 (8)	0.0189 (8)	0.0135 (7)	0.0005 (6)	0.0001 (6)	0.0004 (6)
O1	0.0144 (7)	0.0289 (8)	0.0124 (7)	0.0013 (6)	−0.0005 (5)	−0.0042 (6)
O2	0.0112 (6)	0.0231 (7)	0.0132 (6)	0.0029 (5)	0.0023 (5)	0.0021 (5)
O1W	0.0128 (7)	0.0254 (7)	0.0118 (6)	−0.0028 (5)	0.0006 (5)	−0.0006 (5)
O2W	0.0325 (8)	0.0198 (7)	0.0197 (7)	−0.0101 (6)	0.0145 (6)	−0.0077 (5)

### Geometric parameters (Å, º)

**Table:**

C1—C6	1.391 (3)	C8—H8	0.9300
C1—C2	1.395 (3)	C9—N2	1.312 (2)
C1—N1	1.425 (2)	C9—N1	1.360 (2)
C2—C3	1.387 (3)	C9—H9	0.9300
C2—H2	0.9300	C10—O1	1.250 (2)
C3—C4	1.386 (3)	C10—O2	1.268 (2)
C3—H3	0.9300	Co1—O1W^i^	2.1286 (13)
C4—C5	1.393 (3)	Co1—O1W	2.1286 (13)
C4—C10	1.513 (2)	Co1—O2W	2.0644 (13)
C5—C6	1.395 (2)	Co1—O2W^i^	2.0644 (13)
C5—H5	0.9300	Co1—N2	2.1238 (15)
C6—H6	0.9300	Co1—N2^i^	2.1238 (15)
C7—C8	1.357 (3)	O1W—H1W	0.8307
C7—N1	1.389 (2)	O1W—H2W	0.8296
C7—H7	0.9300	O2W—H4W	0.8479
C8—N2	1.378 (2)	O2W—H3W	0.8402
			
C6—C1—C2	119.67 (16)	O2—C10—C4	118.10 (15)
C6—C1—N1	120.96 (15)	O2W—Co1—O2W^i^	180.0
C2—C1—N1	119.37 (16)	O2W—Co1—N2	89.47 (6)
C3—C2—C1	119.48 (17)	O2W^i^—Co1—N2	90.53 (6)
C3—C2—H2	120.3	O2W—Co1—N2^i^	90.53 (6)
C1—C2—H2	120.3	O2W^i^—Co1—N2^i^	89.47 (6)
C4—C3—C2	121.60 (17)	N2—Co1—N2^i^	180.0
C4—C3—H3	119.2	O2W—Co1—O1W^i^	92.45 (5)
C2—C3—H3	119.2	O2W^i^—Co1—O1W^i^	87.55 (5)
C3—C4—C5	118.53 (16)	N2—Co1—O1W^i^	94.73 (5)
C3—C4—C10	119.50 (16)	N2^i^—Co1—O1W^i^	85.27 (5)
C5—C4—C10	121.91 (16)	O2W—Co1—O1W	87.55 (5)
C4—C5—C6	120.65 (16)	O2W^i^—Co1—O1W	92.45 (5)
C4—C5—H5	119.7	N2—Co1—O1W	85.27 (5)
C6—C5—H5	119.7	N2^i^—Co1—O1W	94.73 (5)
C1—C6—C5	120.01 (16)	O1W^i^—Co1—O1W	180.0
C1—C6—H6	120.0	C9—N1—C7	106.18 (15)
C5—C6—H6	120.0	C9—N1—C1	125.99 (15)
C8—C7—N1	106.30 (16)	C7—N1—C1	127.83 (15)
C8—C7—H7	126.9	C9—N2—C8	106.02 (15)
N1—C7—H7	126.9	C9—N2—Co1	124.04 (13)
C7—C8—N2	109.76 (16)	C8—N2—Co1	129.94 (12)
C7—C8—H8	125.1	Co1—O1W—H1W	127.2
N2—C8—H8	125.1	Co1—O1W—H2W	107.9
N2—C9—N1	111.74 (16)	H1W—O1W—H2W	113.7
N2—C9—H9	124.1	Co1—O2W—H4W	128.4
N1—C9—H9	124.1	Co1—O2W—H3W	114.5
O1—C10—O2	124.93 (16)	H4W—O2W—H3W	110.8
O1—C10—C4	116.95 (15)		

Symmetry code: (i) −*x*+2, −*y*+1, −*z*+2.

### Hydrogen-bond geometry (Å, º)

**Table:**

*D*—H···*A*	*D*—H	H···*A*	*D*···*A*	*D*—H···*A*
O2*W*—H3*W*···O2^ii^	0.84	1.88	2.6745 (17)	157
O2*W*—H4*W*···O2^iii^	0.85	1.86	2.6964 (18)	170
O1*W*—H2*W*···O2^ii^	0.83	2.03	2.8287 (19)	163
O1*W*—H1*W*···O1^iv^	0.83	1.87	2.7014 (18)	177

Symmetry codes: (ii) −*x*+1, −*y*+1, −*z*+2; (iii) −*x*+1, *y*−1/2, −*z*+3/2; (iv) *x*+1, *y*, *z*+1.
